# Single-Stage Immediate Breast Reconstruction with Acellular Dermal Matrix after Breast Cancer: Comparative Study and Evaluation of Breast Reconstruction Outcomes

**DOI:** 10.3390/cancers15225349

**Published:** 2023-11-09

**Authors:** Basilio Dueñas-Rodríguez, Joaquín Navarro-Cecilia, Carolina Luque-López, Belén Sánchez-Andujar, Juan Arsenio Garcelán-Trigo, María Jesús Ramírez-Expósito, José Manuel Martínez-Martos

**Affiliations:** 1 Unit of Breast Pathology, Department of Surgery, Hospital Complex of Jaén, 23007 Jaén, Spain; bdr@quesadasolidaria.org (B.D.-R.); dr.jnavarro@hotmail.com (J.N.-C.); bsa27@hotmail.com (B.S.-A.); 2 Department of Gynecology and Obstetrics, Hospital Complex of Jaén, 23007 Jaén, Spain; carolinaluquelopez@hotmail.com; 3 Department of Radiology, Hospital Complex of Jaén, 23007 Jaén, Spain; juanarsenio@gmail.com; 4 Experimental and Clinical Physiopathology Research Group CTS-1039, Department of Health Sciences, School of Experimental and Health Sciences, University of Jaén, 23071 Jaén, Spain; mramirez@ujaen.es

**Keywords:** skin-sparing mastectomy, immediate reconstruction, breast cancer, biological mesh, BREAST-Q questionnaire

## Abstract

**Simple Summary:**

Breast reconstruction is an important part of the treatment of breast cancer. In modern implant-based immediate breast reconstruction, it is common to use acellular dermal or synthetic meshes in combination with an implant. Here, we describe a prospective cohort study to report the use of a breast implant only versus an implant with a biological mesh in the immediate reconstruction. Breast reconstruction was performed after two types of therapeutic mastectomies (skin-sparing or nipple-sparing), in order to improve aesthetic results and patient wellbeing, and to decrease morbidities and surgical complications. We assessed the patient satisfaction with their breast reconstruction using the BREAST-Q questionnaire, that allows the measuring of the level of satisfaction with the professional, the achieved results and self-perception. We try to contribute in establishment of biological matrices use to achieve the best result in such a delicate moment which is mastectomy therapeutic decision.

**Abstract:**

We evaluate postoperative complications, aesthetic results and satisfaction outcomes in patients with breast cancer after intervening with a skin-sparing or nipple-sparing mastectomy with an immediate prosthetic reconstruction with or without a biological mesh. Patients with multifocal breast cancer, ductal carcinoma in situ with an indication for a mastectomy and cT2 tumors with no response to primary systemic treatment were included, whereas patients aged >75 years, with inflammatory carcinoma, and severe circulatory disorders were excluded. Patients in the control group were reconstructed using a prosthesis, whereas the study group included patients reconstructed using a prosthesis and biological acellular porcine dermal mesh (Strattice™). In both groups, the result was assessed using the BREAST-Q instrument. A total of 51 patients (62 intervened breasts) were included in the study group and 38 patients (41 intervened breasts) in the control group. Implant loss and removal occurred in three patients in the study group (5.9%) and nine patients in the control group (24.3%; *p* = 0.030). Infections appeared in three patients in the study group (4.8%) and three patients in the control group (7.3%; *p* = 1.00). Skin necrosis appeared in 5 patients in the study group (12.2%) and 11 patients in the control group (21.6%; *p* = 0.367). Seroma appeared in five patients in the study group (12.2%) and five patients in the control group (8.1%; *p* = 0.514). The BREAST-Q questionnaire is a comparison between both groups regarding “satisfaction with breasts after surgery” (*p* = 0.026), “sexual well-being after intervention” (*p* = 0.010) and “satisfaction with the information received” (*p* = 0.049). We have noted a statistically significant decrease in implant loss in women receiving an implant with a biological mesh. A higher satisfaction was observed in patients reconstructed using Strattice™, with statistically significant differences in three items.

## 1. Introduction

Breast reconstruction is an important part of the comprehensive treatment of breast cancer. Many advances have been made in this field over the years with the introduction of increasingly less invasive surgical techniques, which achieve excellent results while minimally altering tissues. Among such innovations is the use of a surgical mesh called the acellular dermal matrix (ADM), which has been used in the immediate reconstruction after radical surgery to cover the implant and stabilize its position.

At present, there are numerous biological meshes from allogeneic or xenogeneic sources, derived from products obtained from porcine dermis, bovine pericardium and small bowel submucosa. Acellular dermal matrices from human origin (AlloDerm^®^) have long been used and have been the object of multiple publications, which report good results in clinical use [1]. More recently, there has also been a widespread use of acellular porcine matrices developed using the same principles as meshes from human origin and further processed to remove the alpha-1,3-galactose (a terminal galactose epitope) that is thought to play an important role in xenogeneic rejection [2]. Both matrices are revascularized, recellularized, remodeled and integrated into the host tissue without evidence of encapsulation and contracture [3]. Other types of mesh are also available [4,5].

The surgical technique of mesh-assisted breast reconstruction was first conceived in 2001 by Andrew Salzberg [6] and involves first the release of the lower portion of the pectoralis major muscle and the dissection of the sub pectoral pocket. Subsequently, the ADM sheet is fixed to the inframammary fold and the bottom edge of the pectoralis major, leaving the implant under the pectoral muscle in its upper portion and under the ADM in its lower portion. In this position, the mesh provides an extra layer of coverage and support to the lower pole of the reconstructed breast, without additional muscle raising [7,8] and allowing the surgeon to place the submammary and lateral breast folds in the desired positions.

Various authors have conducted studies analyzing the advantages of using a mesh in breast reconstruction, and while most of them defend the aesthetic results that they provide [9,10,11,12,13,14], their use is not entirely without complications, causing conflicting results in this regard [15]. In their analysis, some authors point out an increase in complications such as infection [16,17] and seroma [16,17,18] when using the ADM. For other authors, these differences do not exist or are not statistically significant [6,9,19].

The purpose of this study is to examine our results in relation to complications arising and patient satisfaction with the aesthetic result in immediate reconstructions in which prosthetic material and a Strattice™ acellular porcine dermal matrix have been used, compared to patients in which only a breast implant was used.

## 2. Materials and Methods

### 2.1. Patient Population

This study has included all the patients who underwent a skin-sparing mastectomy or a nipple-sparing mastectomy with immediate reconstruction. The control group patients underwent reconstruction using only an Allergan™ Natrelle™ 410 (Allergan, Irvine, CA, USA) cohesive silicone gel anatomical breast implant, whereas the study group patients underwent breast reconstruction with an acellular dermal matrix of porcine origin (Strattice LifeCell Corp., Branchburg, NJ, USA), in addition to the implant.

### 2.2. Inclusion and Exclusion Criteria

Inclusion criteria were multifocal infiltrating breast cancer, cT2 tumors with no response to primary systemic treatment and ductal carcinomas in situ with indication for a mastectomy (large (>4 cm) multifocal tumors with diffuse micro-calcifications, palpable masses or inability to obtain negative margins in conservative surgery). Exclusion criteria were age >75 years and inflammatory carcinoma. The surgical indication was as cancer treatment for breast cancer diagnosis. Period: from October 2011 to October 2015.

### 2.3. Surgical Technique

**Figure 1:**
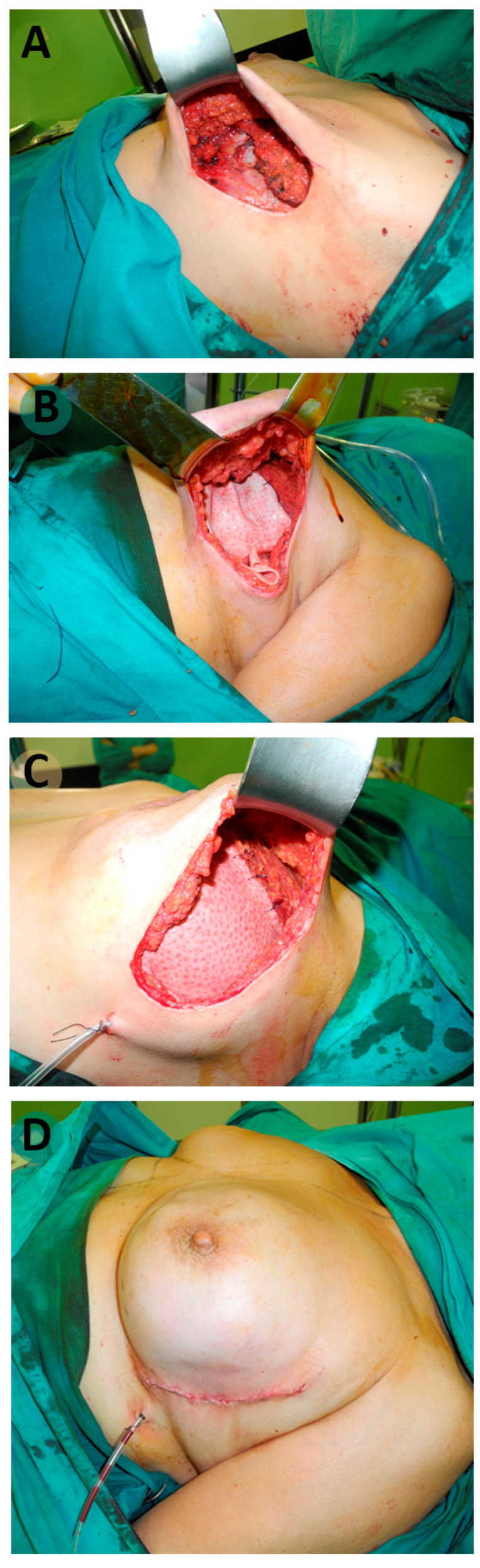
(**A**–**D**) Nipple-sparing mastectomy by lateral approach. Biological mesh Strattice™ fixed to the pectoralis major muscle edge and then to the inframammary fold. Anatomical implant placement. Direct-to-implant breast reconstruction.

Regarding the surgical technique, all implants were placed in the retro-muscular position. In cases using an ADM, the implant was covered by the pectoralis major muscle in the upper portion and by the ADM at the lower portion. The ADM was treated as directed by the manufacturer. For reconstructions without the matrix, a partial muscle coverage of the implant was performed by unseating the lower edge of the pectoralis major muscle (Figure 1). The most commonly used incisions were the anterior axillary line and the inferior hemiareolar line. In some cases, they were associated in the same surgical time to skin patterns of mastopexy/reduction (vertical or inverted T patterns) or even to skin perforators flaps. The approach for axillary surgery, selective sentinel node biopsy (SLNB) or lymphadenectomy was performed using a mammary incision. Contralateral breast symmetrization was performed in the same surgical procedure in cases where it was necessary.

All patients were left with a suction drain placed between the breast implant and the pectoralis major muscle at its distal end and between the mesh and subcutaneous tissue in the proximal portion of the drain; a second drain was placed in the armpit in the case of axillary lymphadenectomy.

Antibiotic prophylaxis was initiated before surgery, maintaining it intravenously until discharge and then completed orally for 7 days if no complications appeared.

Immediately after the intervention, we recommended the use of a special postsurgical compression bra to ensure light pressure with uniform breast distribution to minimize dead spaces but without compromising vascularization of the area.

### 2.4. Study of Complications

In both groups of patients undergoing immediate reconstruction with or without an ADM, the rate of complications (skin necrosis, loss of NAC (Nipple-Areolar Complex), infection, seroma, hematoma and extrusion of the prosthesis) was assessed, as well as whether they had received prior chemotherapy (CT) or radiation therapy (RT) and other factors that could affect the rate of complications. The results observed in both groups were statistically compared using the Chi-square test or Fisher’s test, as they are all qualitative variables. Differences were considered statistically significant for a value of *p* < 0.05.

### 2.5. Patient-Reported Outcome Measure

The BREAST-Q (Memorial Sloan Kettering Cancer Center and The University of British Columbia ©2006, all rights reserved) instrument meets breast reconstructive surgery quality standards in terms of validation and development. This questionnaire is designed to measure the impact of breast reconstruction in quality of life and satisfaction from the patient’s viewpoint. The BREAST-Q reconstruction module consists of 9 scales. Each scale has three to five items using a Likert scale. The score of each scale is subsequently transformed into a 100 points scale. Thus, each scale shows a score from 0 (very unsatisfied) to 100 (very satisfied). The scales have good internal consistency (Cronbach’s alpha between 0.88 and 0.97). The BREAST-Q reconstruction module is divided into multiple independent scales: satisfaction with breasts (16 items), satisfaction with the outcome (7 items), psychosocial well-being (10 items), physical well-being (16 items) and sexual well-being (6 items). For each scale, the responses to the items are added and transformed into a scale ranging from 0 to 100. Higher scores indicate higher satisfaction or quality of life.

## 3. Results

### 3.1. Study Population

A total of 103 immediate reconstructions after mastectomy were performed in the 79 patients included in the study, with an age range of 31–75 years and an average of 48.9 years. In 41 cases, the reconstruction was carried out using only the implant, and in 62 reconstructions, the Strattice™ mesh was used in addition to the breast implant. In 31 (30.1%) reconstructions, a skin-saving mastectomy was performed, and in 72 (69.9%) cases, a skin-saving mastectomy and an NAC were used.

### 3.2. Study Design

This was a cohort study, with two population prospective cohorts, and we sequentially collected data from each one: from the beginning of the activity (single plastic surgeon) of immediate reconstruction using implants without mesh and later with ADM when the use was financed.

### 3.3. Between-Groups Complication Comparison

Upon analyzing the two groups of patients separately, we found that both groups were homogeneous in terms of age, diabetes, smoking and prior treatment with CT and/or RT (Table 1).

When comparing the complications in both groups, we found no statistically significant differences for seroma, infection, hematoma, skin necrosis and loss of NAC (Table 2). However, implant loss was more frequent in the reconstructions in which no Strattice™ mesh was used (24.3% vs. 5.9%) (*p* = 0.030). The implants used were Allergan Natrelle 410 (cohesive gel anatomical prosthesis), with a median of 410 g in the right breast (range: 160–685 g) and 375 g in the left breast (range: 195–685 g).

The relative risk of exposure of the biological mesh against the effect of having an extrusion is 0.242 with 95% CI (0.070 to 0.832). This indicates that the biological mesh is a protective factor in the presence of extrusion, and therefore, patients implanted with a biological mesh had a reduced presence of extrusions by 75.8% (1–0.242 = 0.758) compared to the group not implanted with a biological mesh. The number of necessary to treat patients (NNT) with a biological mesh to prevent an extrusion is 3.31.

### 3.4. Measuring Patient Outcomes

The results of the BREAST-Q satisfaction survey are shown in Table 3. The total percentage of participation was 79.78% (29 patients completed the survey out of the 38 that made up the group that received no biological mesh (76.32%) and 42 out of the 51 patients reconstructed using the mesh and implant (82.35%)).

## 4. Discussion

### 4.1. Suitable Mesh

Biological or synthetic meshes can be used in various circumstances, for example, in immediate implant-based reconstruction after an oncological mastectomy or in risk-reducing surgery. Their position is prepectoral or partial subpectoral. The most common use is in immediate subpectoral reconstruction, acting as an extension of the pectoralis major muscle. In this way, the mesh is fixed to the inferolateral portion of the muscle and to the submammary fold, to fill the space between the muscle and the fascia, and thus recreate the lower pole of the breast [20].

In prepectoral reconstruction, the precontoured mesh allows for covering the implant, avoiding its displacement and giving greater thickness to the reconstruction [21].

Currently, there is no clear consensus or evidence to select one type of mesh or another, and studies are recommended to evaluate the long-term impact of its use in the clinic and the level of patient satisfaction [22,23].

After reviewing the literature, the Strattice™ mesh of porcine origin and subpectoral position were chosen for its qualities: it is easier to use as it does not require rehydration, it can be ready after two minutes of saline rinsing, it is available in large sizes, it has a good thickness for coating, it can be stored at room temperature, and it has no polarity.

For the intraoperative management of the mesh, antibiotic prophylaxis, antiseptic preparation of the skin, change of gloves for its handling, measures in the operating room to avoid contamination, avoiding the transfer of personnel, control of the patient’s temperature, intracavitary drains and washing of the pocket are recommended.

### 4.2. Complications after Radiotherapy

In reconstructions after a mastectomy, neoadjuvant and adjuvant therapy, and especially after radiation therapy, the number of complications and loss of implants increases. Thus, Krueger et al. [24] observed a 68% complication rate in irradiated patients compared to 31% in non-irradiated patients, with a higher percentage of implant loss. Cordeiro and McCarthy [25] also report a greater absolute number of complications in irradiated versus non-irradiated patients (11.7% vs. 5.6%), with a higher percentage of infection, implant loss, necrosis and seroma. So, we consider that it is important that both groups of patients were homogeneous with regard to the history of having received RT and/or CT in order to study the rate of complications that can be attributed to the use or not of ADM, as these factors could falsify the interpretation of the results [26].

### 4.3. Analysis of Results

Analyzing our results, we have not found a significant increase in the incidence of seromas, hematomas, infections or skin necrosis, including loss of the NAC, when performing reconstructions with an ADM vs. the cases without an ADM. These results are comparable to those of other series published, which did not find a significant increase either in the incidence of complications when using an ADM in breast reconstruction [6,9,10,19,27]. There is controversy about this because some works in the literature report a significant increase in the incidence of postoperative complications with the use of the ADM. Some refer to the increase in the rate of seroma, infection, necrosis and even implant loss [27], while others only report a significant increase in the rate of seroma [16,18,28] and infections [16,17,29,30,31,32]. The cause of such different results is unclear. Some authors [2,13,33,34,35,36,37] suggest that most of the studies conducted on the effect of an ADM in the postoperative results were made with AlloDerm^®^, which is a product that has been sold for longer, and only more recently, a series has been published with the use of other ADM, both from human and porcine origin, and although the preparation is similar in removing the cellularity of the tissue and infectious organisms, there may be subtle unpublished differences in the processing and storage of these products, causing an inflammatory response with a higher influence in the formation of seroma that could also be related to the increase in other complications such as infections or extrusions of the implant [34]. Reference is also made to important immunological differences between the matrices of human or non-human origin that can influence the rate of complications, preventing extrapolation of results from different studies [14,35,38,39,40]. Other works consider that the fact that there is not a unanimous criterion defining the concept of seroma or infection could also influence the different results, since clinical signs can be interpreted differently. Brzezienski [41] concludes that it is necessary to define the concept of seroma on immediate reconstructions using an ADM and categorize it to discuss its impact and to propose solutions to reduce its incidence. It is also necessary to unify the definition of infection because it can be confused with the Red Breast Syndrome (self-limiting rash attributable to recellularized ADM), which may mistakenly raise their statistical incidence. Red Breast Syndrome was observed in a case in our series, and we managed to differentiate it from an infectious process.

### 4.4. Lower Rate of Loss Implant with ADM Use

In our study, the extrusion of the implant when using Strattice™ in immediate reconstruction occurred in 5.9% of cases, a similar result to that published in other series using an ADM [18,28,29,42]. In addition, we have obtained a statistically significant decrease in the incidence of extrusion when compared to a reconstruction without the use of an ADM. Meta-analysis have been performed with conflicting results in this regard. Kim et al. [42] and Ho et al. [43] reported a higher loss of the implant with an ADM; however, Sbitany et al. [44] found no differences when reconstruction was performed with an ADM or not. In our study, the high percentage of extrusions in the group of patients reconstructed only with prosthetic material may be due to the fact that in all cases, radical surgery was with a skin-sparing mastectomy or skin and NAC and immediate reconstruction with an implant in all cases, also including vertical skin or inverted T patterns in the same surgical procedure, thus increasing the risk of skin necrosis and exposure of the prosthesis, which explains the drastic reduction in the number of lost implants when using Strattice™ to completely cover the implant.

### 4.5. Higher Level of Satisfaction with ADM Breast Reconstruction

Breast reconstruction has evolved to give better results, not only for aesthetics outcomes, but also with lower morbidities. Every patient undergoes secondary changes in a different way; physical changes affect their body image and may involve stress and anxiety, hair loss, fatigue, limitation of upper limb function, etc.

Immediate breast reconstruction has been demonstrated to improve patient satisfaction and decrease the incidence of postoperative depression.

This study evaluated satisfaction outcomes with breast reconstruction, psychosocial wellness and sexual wellbeing, comparing patients with immediate reconstruction using an ADM and an implant or only an implant.

Although there are limited data available on patient-reported outcomes in Acellular Dermal Matrix (ADM)-assisted breast reconstruction [45], they show high levels of satisfaction with the Strattice™ cosmetic outcomes.

### 4.6. Limitations of the Study

We present a moderate-sized prospective study comparing outcomes between patients undergoing single-stage implant-based reconstruction with partial submuscular/partial Strattice™ coverage to those undergoing reconstruction with partial muscle coverage alone; although, our study population is only breast cancer affected.

We think a single center and single plastic surgeon sample is an advantage because it removes between-center and between-surgeon variability.

## 5. Conclusions

There is a benefit of using Strattice™ in immediate reconstruction after a mastectomy because although it does not diminish the incidence of some immediate postoperative complications requiring surgical intervention, the matrix that has a better tolerance to exposure, decreases the risk of implant loss and also decreases the risk of breast reconstruction failure. Likewise, satisfaction surveys after the intervention are also statistically significant in favor of the use of biological meshes with regard to sexual well-being, satisfaction with breasts and satisfaction with the information received.

## Figures and Tables

**Table 1:** Comparative table for the case and control groups.

	Mesh (*n* = 51)	No Mesh (*n* = 38)	*p*-Value	Test
Age	49.50 (1.49)	47.93 (1.89)	0.521	Student’s *t*-test
Diabetes	2 (3.92%)	0 (0%)	0.505	Fisher’s exact test
Smoking	6 (11.76%)	1 (2.63%)	0.231	Fisher’s exact test
Radiotherapy RT	16 (31.4%)	10 (27.0%)	0.838	Chi-square with Yates correction
Pre-operative	5 (31.3%)	3 (30.0%)	1.000	Fisher’s exact test *
Post-operative	11 (68.8%)	7 (70.0%)		
Neoadjuvant Chemotherapy	29 (56.9%)	18 (48.6%)	0.585	Chi-square with Yates correction
Adjuvant Chemotherapy	15 (29.4%)	7 (18.9%)	0.383	Chi-square with Yates correction
BRCA1	7 (13.7%)	0 (0%)	0.020	Fisher’s exact test
BRCA2	2 (3.9%)	1 (2.7%)	1	Fisher’s exact test

Age is shown as mean ± SE and qualitative variables are shown as frequencies and percentages. * These values are referred to the 26 patients receiving RT.

**Table 2:** Frequency of major complications in both groups.

	Mesh (*n* = 62)	No Mesh (*n* = 41)	*p*-Value
Skin necrosis	11 (21.6%)	5 (12.2%)	0.367 (Chi-square with Yates correction)
Seroma	5 (8.1%)	5 (12.2%)	0.514 (Fisher’s exact test)
Extrusion of the implant	3 (5.9%)	9 (24.3%)	0.030 (Fisher’s exact test)
Total or partial loss of NAC	3 (4.8%)	4 (9.8%)	0.432 (Fisher’s exact test)
Infection	3 (4.8%)	3 (7.3%)	0.680 (Fisher’s exact test)
Hematoma	5 (8.1%)	3 (7.3%)	1.000 (Fisher’s exact test)

**Table 3:** Descriptions of BREAST-Q variables according to treatment group.

Variable	Group	N	Average	SD	Typical Error	Minimum	Maximum	Rank	Median
Satisfaction with Breasts pre	No Mesh	29	58.66	22.283	4.138	0	100	100	63.00
	Mesh	42	63.26	21.226	3.275	0	100	100	63.00
	Total	71	61.38	21.626	2.567	0	100	100	63.00
PsychoSocial Wellbeing pre	No Mesh	29	67.66	16.715	3.104	37	100	63	67.00
	Mesh	42	74.60	17.576	2.712	36	100	64	77.50
	Total	71	71.76	17.450	2.071	36	100	64	70.00
Physical Wellbeing Chest pre	No Mesh	29	61.86	16.803	3.120	31	100	69	60.00
	Mesh	41	64.34	15.244	2.381	25	100	75	63.00
	Total	70	63.31	15.837	1.893	25	100	75	62.00
Physical Wellbeing Abdomen pre	No Mesh	28	68.07	21.022	3.973	25	100	75	72.00
	Mesh	40	75.70	22.869	3.616	14	100	86	83.00
	Total	68	72.56	22.289	2.703	14	100	86	77.50
Sexual Wellbeing pre	No Mesh	26	48.58	20.379	3.997	16	100	84	46.00
	Mesh	41	62.73	22.808	3.562	16	100	84	60.00
	Total	67	57.24	22.823	2.788	16	100	84	54.00
Satisfaction with Breasts post	No Mesh	26	54.46	17.591	3.450	20	78	58	56.00
	Mesh	41	66.22	16.044	2.506	40	100	60	65.00
	Total	67	61.66	17.509	2.139	20	100	80	62.00
Satisfaction with Outcome post	No Mesh	25	77.96	21.532	4.306	35	100	65	75.00
	Mesh	40	82.90	17.666	2.793	35	100	65	80.50
	Total	65	81.00	19.233	2.386	35	100	65	75.00
PsychoSocial Wellbeing post	No Mesh	26	71.54	18.862	3.699	41	100	59	70.00
	Mesh	39	79.26	18.113	2.900	26	100	74	49.00
	Total	65	76.17	18.663	2.315	26	100	74	76.00
Sexual Wellbeing post	No Mesh	23	49.91	21.753	4.536	16	100	84	49.00
	Mesh	38	64.50	24.380	3.955	0	100	100	60.00
	Total	61	59.00	24.307	3.112	0	100	100	57.00
Physical Wellbeing Chest post	No Mesh	26	56.92	15.226	2.986	47	100	53	50.00
	Mesh	35	51.29	3.777	0.639	50	68	18	50.00
	Total	61	53.69	10.611	1.359	47	100	53	50.00
Physical Wellbeing Abdomen post	No Mesh	Not evaluated because they do not apply to operated patients							
	Mesh								
	Total								
Satisfaction with Nipples post	No Mesh	Not evaluated because they do not apply to operated patients							
	Mesh								
	Total								
Satisfaction with Information post	No Mesh	23	65.13	19.485	4.063	25	100	75	60.00
	Mesh	38	75.03	20.346	3.301	22	100	78	72.50
	Total	61	71.30	20.442	2.617	22	100	78	69.00
Surgeon post	No Mesh	25	88.28	19.208	3.842	31	100	69	100.00
	Mesh	38	92.34	13.869	2.250	29	100	71	100.00
	Total	63	90.73	16.175	2.038	29	100	71	100.00
Medical Staff post	No Mesh	25	99.36	3.200	0.640	84	100	16	100.00
	Mesh	40	96.20	8.489	1.342	74	100	26	100.00
	Total	65	97.42	7.082	0.878	74	100	26	100.00
Office Staff post	No Mesh	24	99.00	3.502	0.715	85	100	15	100.00
	Mesh	37	95.08	12.698	2.088	43	100	57	100.00
	Total	61	96.62	10.255	1.313	43	100	57	100.00

## Data Availability

The data presented in this study are available on request from the corresponding author.
